# Supplementary material for: Characterization of the Effects of a Novel Probiotic on Salmonella Colonization of a Piglet-Derived Intestinal Microbiota Using Improved Bioreactor
Source: Animals (Basel). 2024 Mar 2;14(5):787. doi: 10.3390/ani14050787 (PMC10930937; doi:10.3390/ani14050787)
Supplement: Supplementary file 1 [file animals-14-00787-s001.zip › animals-2855905-supplementary.pdf]

Supplementary Materials:

Supplementary Table S1: PCR primer's sequence

Primers'name	Primer's sequence
15FP1-CS1F (forward)	5′-ACACTGACGACATGGTTC-TACAGTGCCAGCMGCCGCGG-TAA-3′
806RP1-CS2R (reverse)	5′-TACGGTAGCAGAGACTT-GGTCTGGAC-TACHVGGGTWTCTAAT-3′

Supplementary Table S2: p values returned by the pairwise ADONIS test for Figure 2 comparison of each treatment at each timepoint.

Conditions	CTL 48h	Daily 48h	CTL 72h	Daily 72h	CTL 96h	Daily 96h	CTL 120h	Daily 120h
Continue 48h	p>0.05	p>0.05	X	X	X	X	X	X
Daily 48h	p>0.05	X	X	X	X	X	X	X
Continue 72h	X	X	0.001	0.038	X	X	X	X
Daily 72h	X	X	0.022	X	X	X	X	X
Continue 96h	X	X	X	X	0.001	0.002	X	X
Daily 96h	X	X	X	X	0.042	X	X	X
Continue 120h	X	X	X	X	X	X	0.001	0.008
Daily 120h	X	X	X	X	X	X	0.032	X

h:hours; x: no comparison made

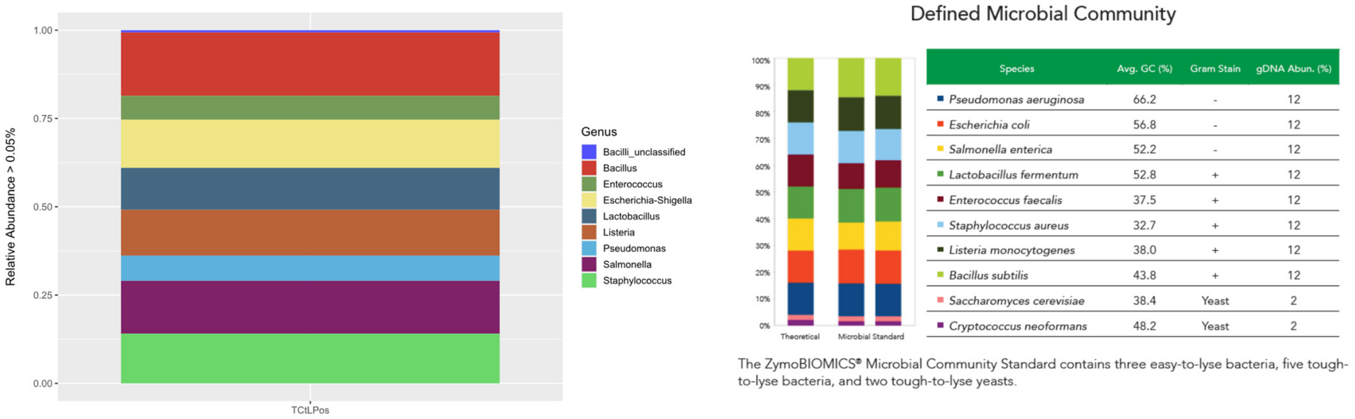

Supplementary Figure S1: Positive control composition according to manufacturer description.

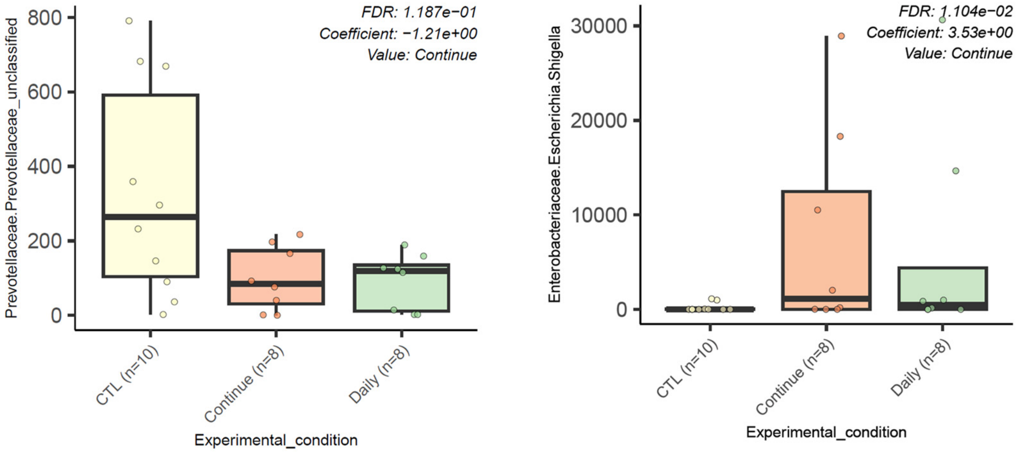

Supplementary Figure S2: Significant genera at T48 for both treatment in comparison with the control analyzed by MaAslin2

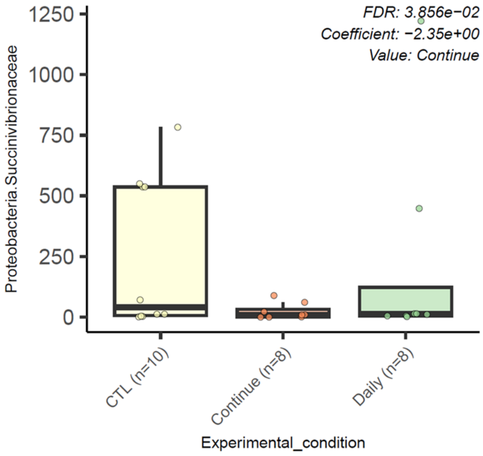

Supplementary Figure S3: Significant family T48 for both treatment in comparison with the control analyzed by MaAslin2

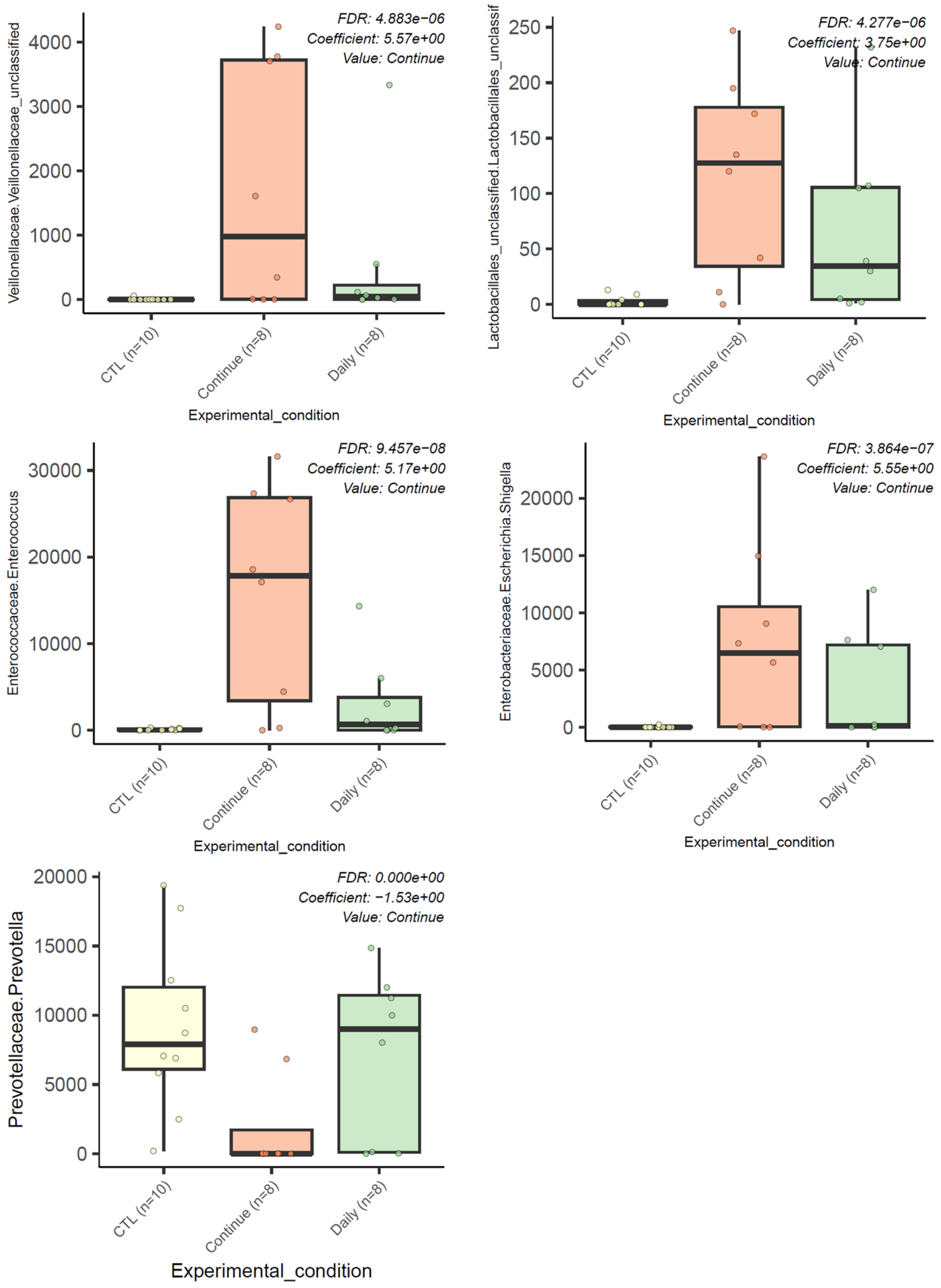

FDR: 4.883e−06
Coefficient: 5.57e+00
Value: Continue
Veillonellaceae.Veillonellaceae_unclassified
4000
3000
2000
1000
0
CTL (n=10)
Continue (n=8)
Daily (n=8)
Experimental_condition
FDR: 4.277e−06
Coefficient: 3.75e+00
Value: Continue
Lactobacillales_unclassified.Lactobacillales_unclassif
250
200
150
100
50
0
CTL (n=10)
Continue (n=8)
Daily (n=8)
Experimental_condition
FDR: 9.457e−08
Coefficient: 5.17e+00
Value: Continue
Enterococcaceae.Enterococcus
30000
20000
10000
0
CTL (n=10)
Continue (n=8)
Daily (n=8)
Experimental_condition
FDR: 3.864e−07
Coefficient: 5.55e+00
Value: Continue
Enterobacteriaceae.Escherichia.Shigella
20000
15000
10000
5000
0
CTL (n=10)
Continue (n=8)
Daily (n=8)
Experimental_condition
FDR: 0.000e+00
Coefficient: −1.53e+00
Value: Continue
Prevotellaceae.Prevotella
20000
15000
10000
5000
0
CTL (n=10)
Continue (n=8)
Daily (n=8)
Experimental_condition

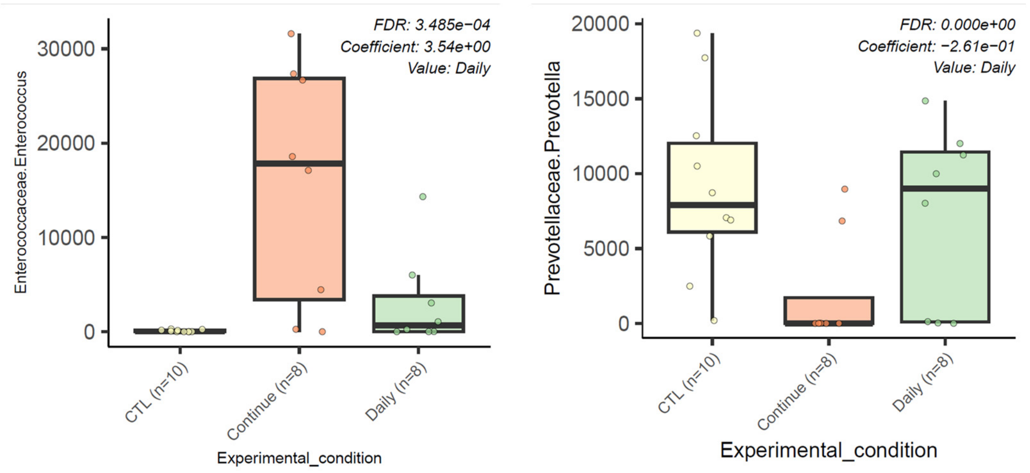

Supplementary Figure S4: Significant genera T72 for both treatment in comparison with the control analyzed by MaAslin2

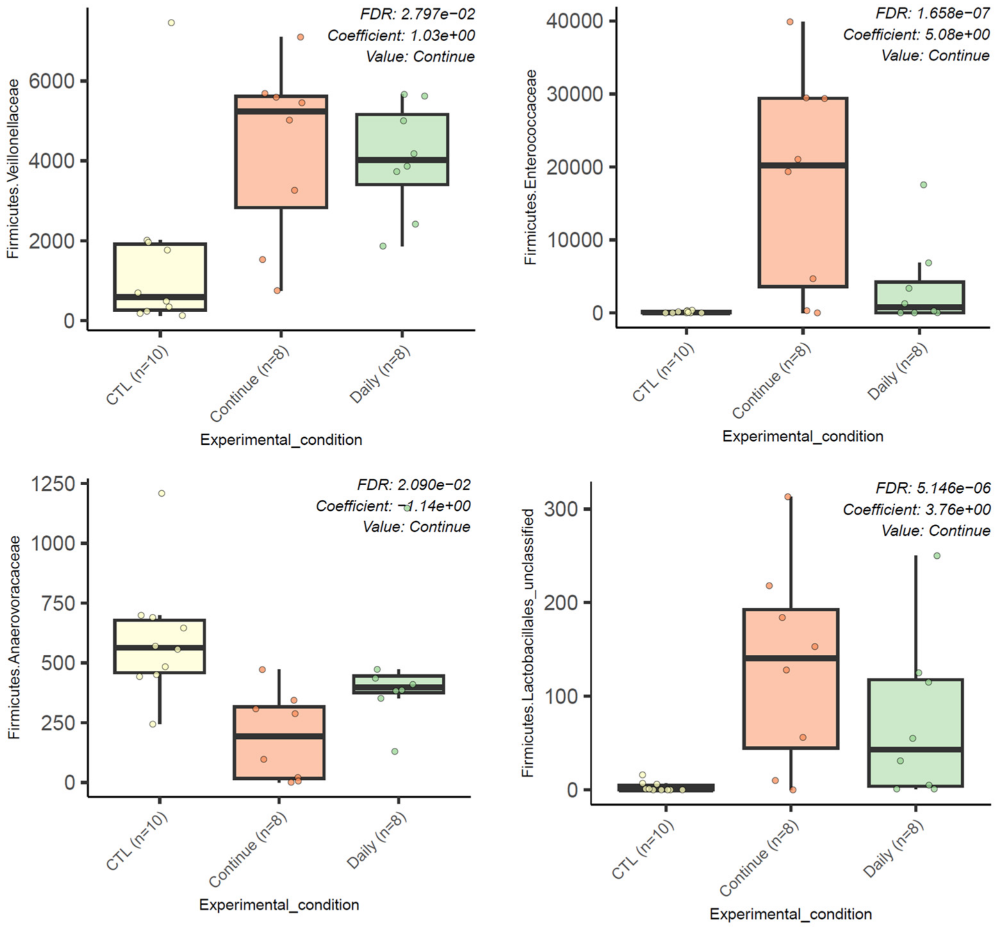

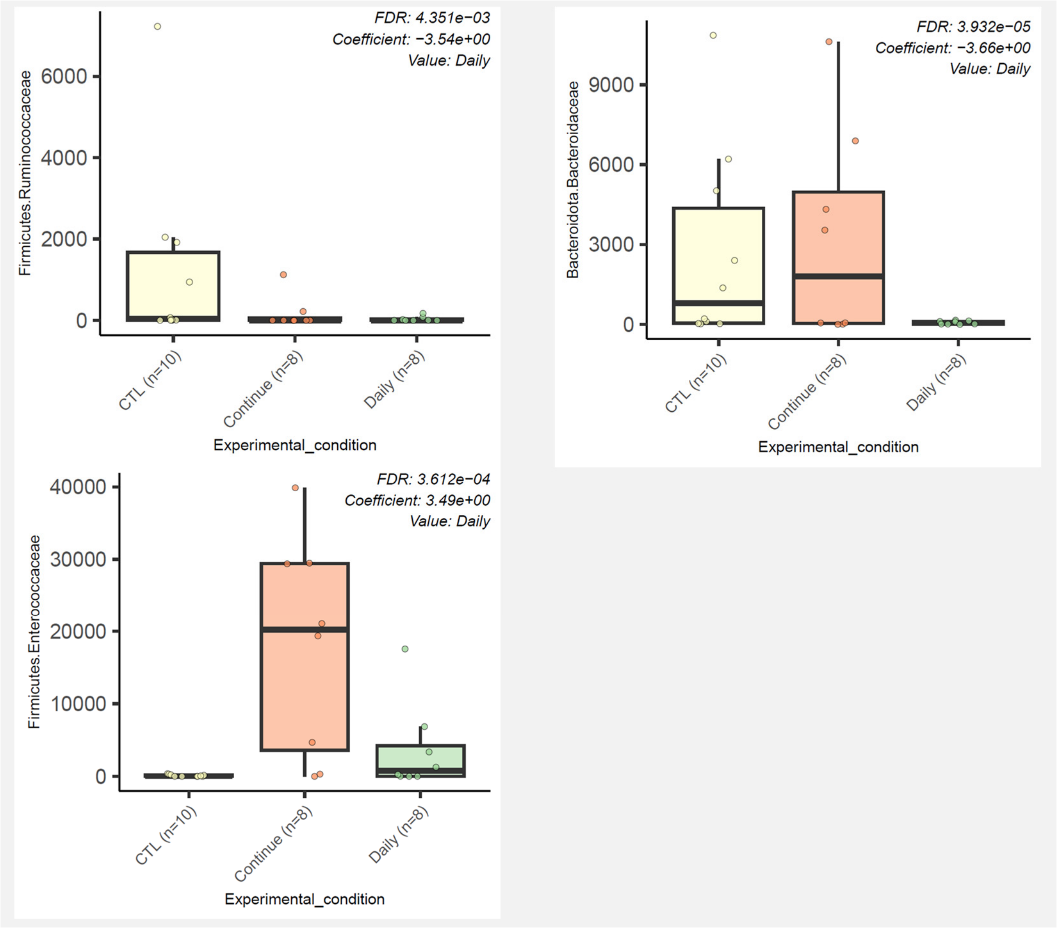

Supplementary Figure S5: Significant family T72 for both treatment in comparison with the control analyzed by MaAslin2

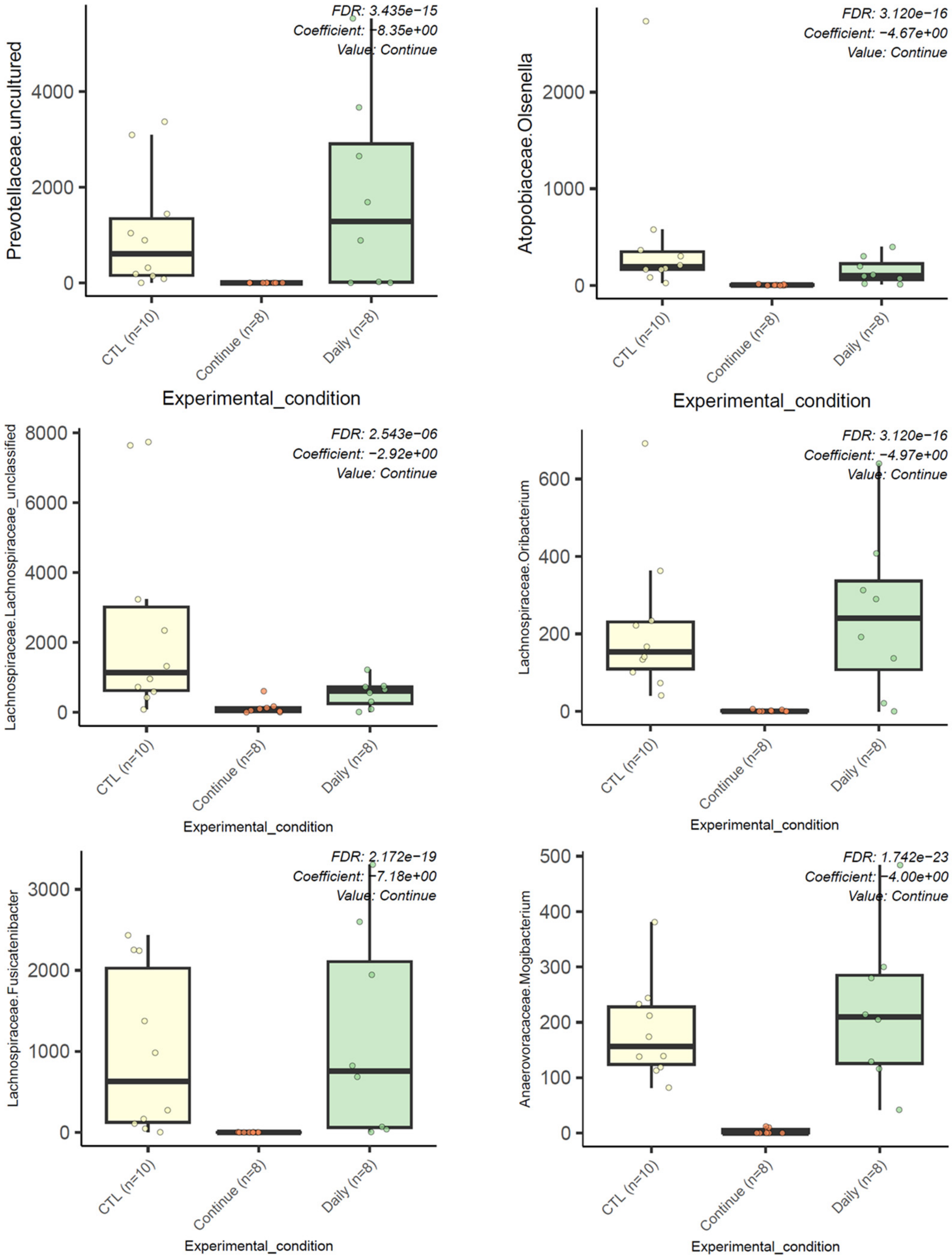

FDR: 3.435e−15
Coefficient: −8.35e+00
Value: Continue
Prevotellaceae.uncultured
4000
2000
0
CTL (n=10)
Continue (n=8)
Daily (n=8)
Experimental_condition
FDR: 3.120e−16
Coefficient: −4.67e+00
Value: Continue
Atopobiaceae.Olsenella
2000
1000
0
CTL (n=10)
Continue (n=8)
Daily (n=8)
Experimental_condition
FDR: 2.543e−06
Coefficient: −2.92e+00
Value: Continue
Lachnospiraceae.Lachnospiraceae_unclassified
8000
6000
4000
2000
0
CTL (n=10)
Continue (n=8)
Daily (n=8)
Experimental_condition
FDR: 3.120e−16
Coefficient: −4.97e+00
Value: Continue
Lachnospiraceae.Oribacterium
600
400
200
0
CTL (n=10)
Continue (n=8)
Daily (n=8)
Experimental_condition
FDR: 2.172e−19
Coefficient: −7.18e+00
Value: Continue
Lachnospiraceae.Fusicatenibacter
3000
2000
1000
0
CTL (n=10)
Continue (n=8)
Daily (n=8)
Experimental_condition
FDR: 1.742e−23
Coefficient: −4.00e+00
Value: Continue
Anaerovoracaceae.Mogibacterium
500
400
300
200
100
0
CTL (n=10)
Continue (n=8)
Daily (n=8)
Experimental_condition

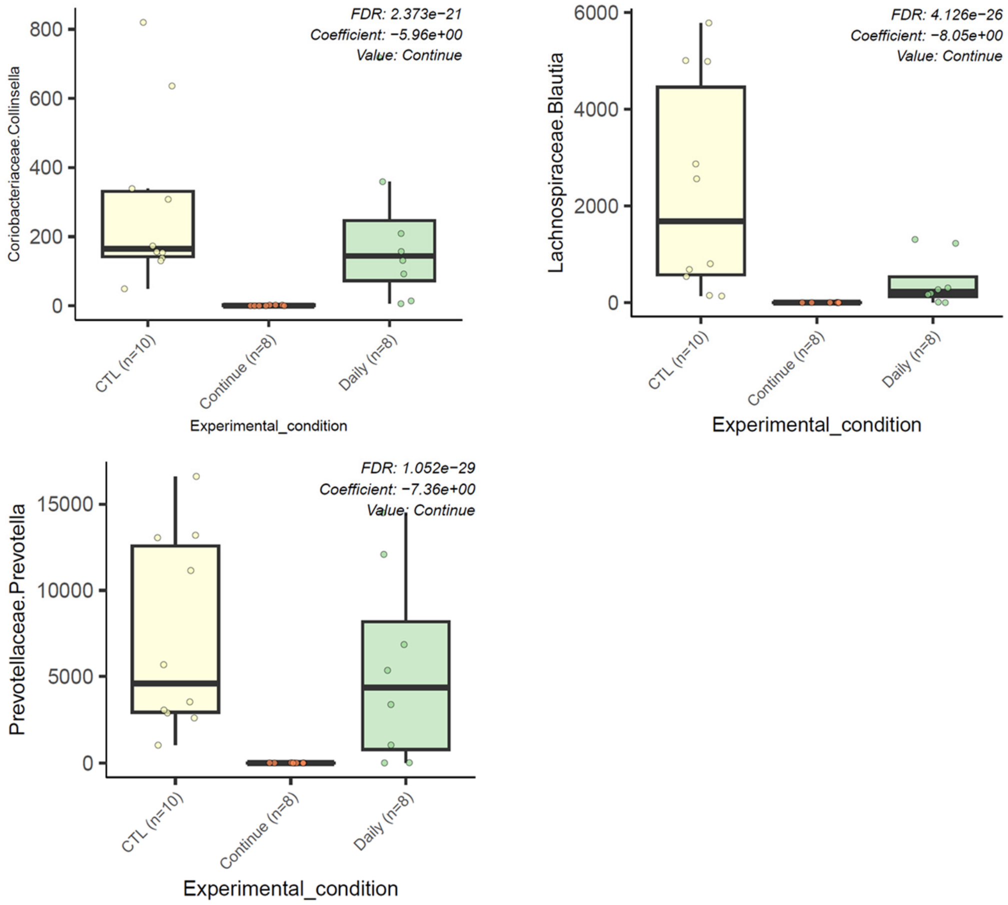

Supplementary Figure S6: Significant genera T96 for both treatment in comparison with the control analyzed by MaAslin2

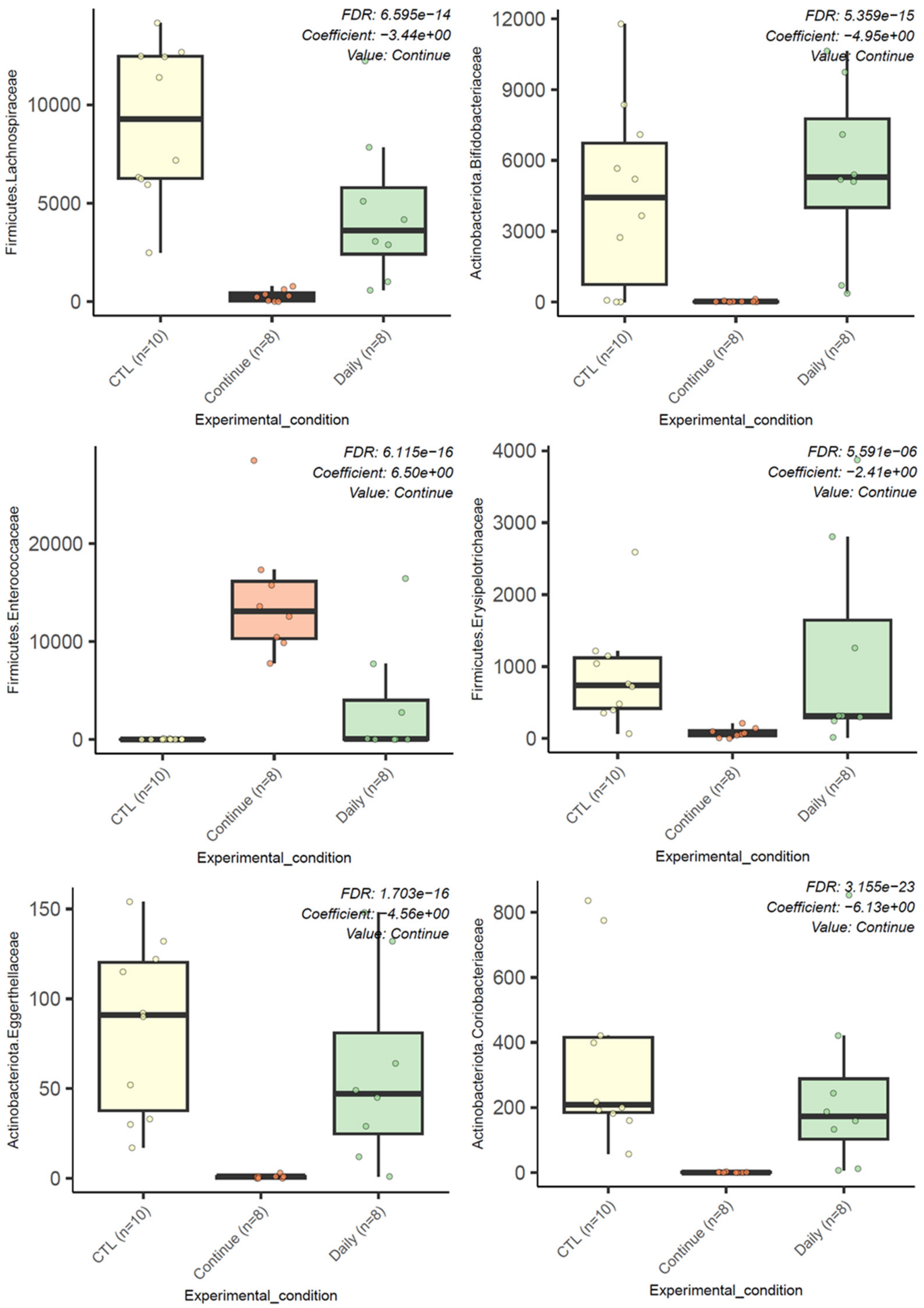

FDR: 6.595e−14
Coefficient: −3.44e+00
Value: Continue
Firmicutes.Lachnospiraceae
10000
5000
0
CTL (n=10)
Continue (n=8)
Daily (n=8)
Experimental_condition
FDR: 5.359e−15
Coefficient: −4.95e+00
Value: Continue
Actinobacteriota.Bifidobacteriaceae
12000
9000
6000
3000
0
CTL (n=10)
Continue (n=8)
Daily (n=8)
Experimental_condition
FDR: 6.115e−16
Coefficient: 6.50e+00
Value: Continue
Firmicutes.Enterococcaceae
20000
10000
0
CTL (n=10)
Continue (n=8)
Daily (n=8)
Experimental_condition
FDR: 5.591e−06
Coefficient: −2.41e+00
Value: Continue
Firmicutes.Erysipelotrichaceae
4000
3000
2000
1000
0
CTL (n=10)
Continue (n=8)
Daily (n=8)
Experimental_condition
FDR: 1.703e−16
Coefficient: −4.56e+00
Value: Continue
Actinobacteriota.Eggerthellaceae
150
100
50
0
CTL (n=10)
Continue (n=8)
Daily (n=8)
Experimental_condition
FDR: 3.155e−23
Coefficient: −6.13e+00
Value: Continue
Actinobacteriota.Coriobacteriaceae
800
600
400
200
0
CTL (n=10)
Continue (n=8)
Daily (n=8)
Experimental_condition

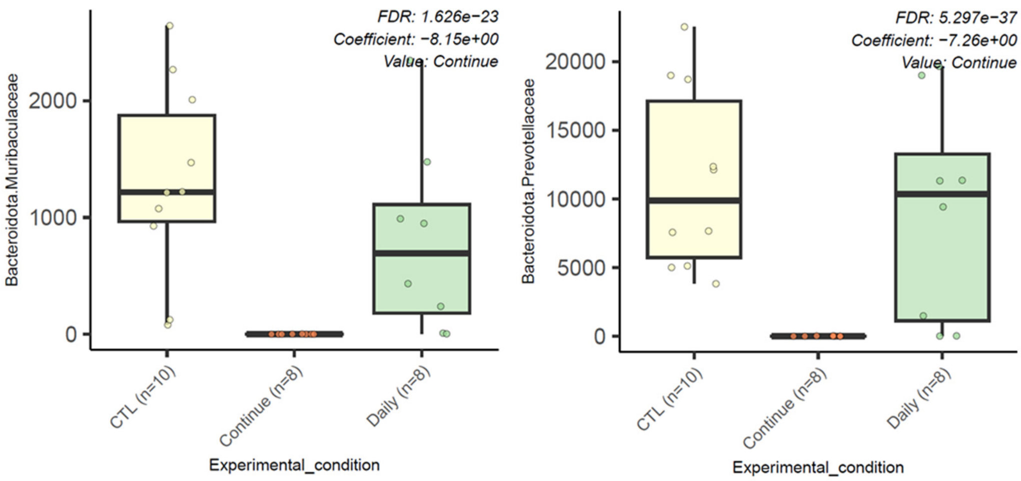

Supplementary Figure S7: Significant family T96 for both treatment in comparison with the control analyzed by MaAslin2

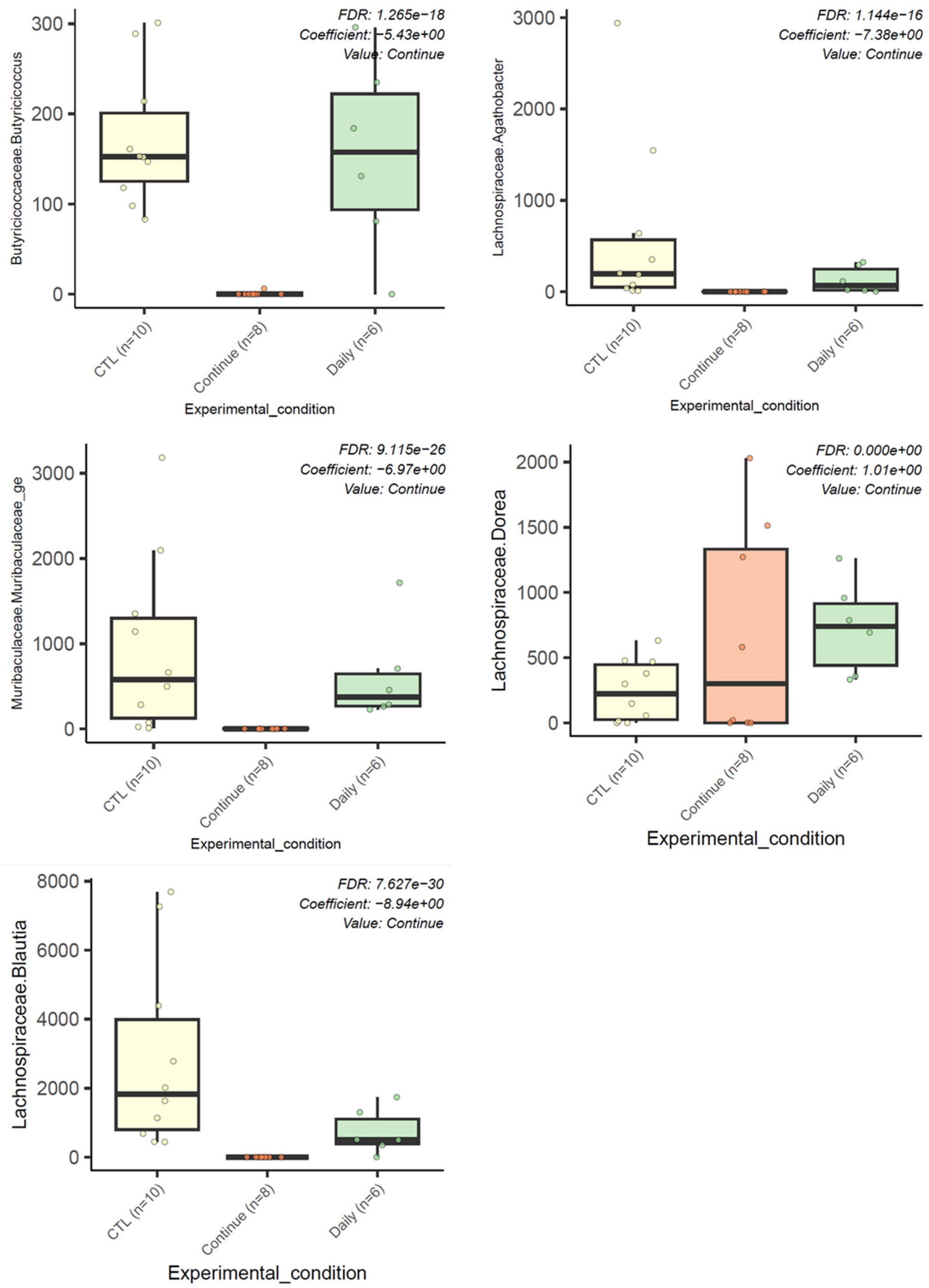

FDR: 1.265e−18
Coefficient: −5.43e+00
Value: Continue
Butyricicoccaceae.Butyricicoccus
300
200
100
0
CTL (n=10)
Continue (n=8)
Daily (n=6)
Experimental_condition
FDR: 1.144e−16
Coefficient: −7.38e+00
Value: Continue
Lachnospiraceae.Agathobacter
3000
2000
1000
0
CTL (n=10)
Continue (n=8)
Daily (n=6)
Experimental_condition
FDR: 9.115e−26
Coefficient: −6.97e+00
Value: Continue
Muribaculaceae.Muribaculaceae_ge
3000
2000
1000
0
CTL (n=10)
Continue (n=8)
Daily (n=6)
Experimental_condition
FDR: 0.000e+00
Coefficient: 1.01e+00
Value: Continue
Lachnospiraceae.Dorea
2000
1500
1000
500
0
CTL (n=10)
Continue (n=8)
Daily (n=6)
Experimental_condition
FDR: 7.627e−30
Coefficient: −8.94e+00
Value: Continue
Lachnospiraceae.Blautia
8000
6000
4000
2000
0
CTL (n=10)
Continue (n=8)
Daily (n=6)
Experimental_condition

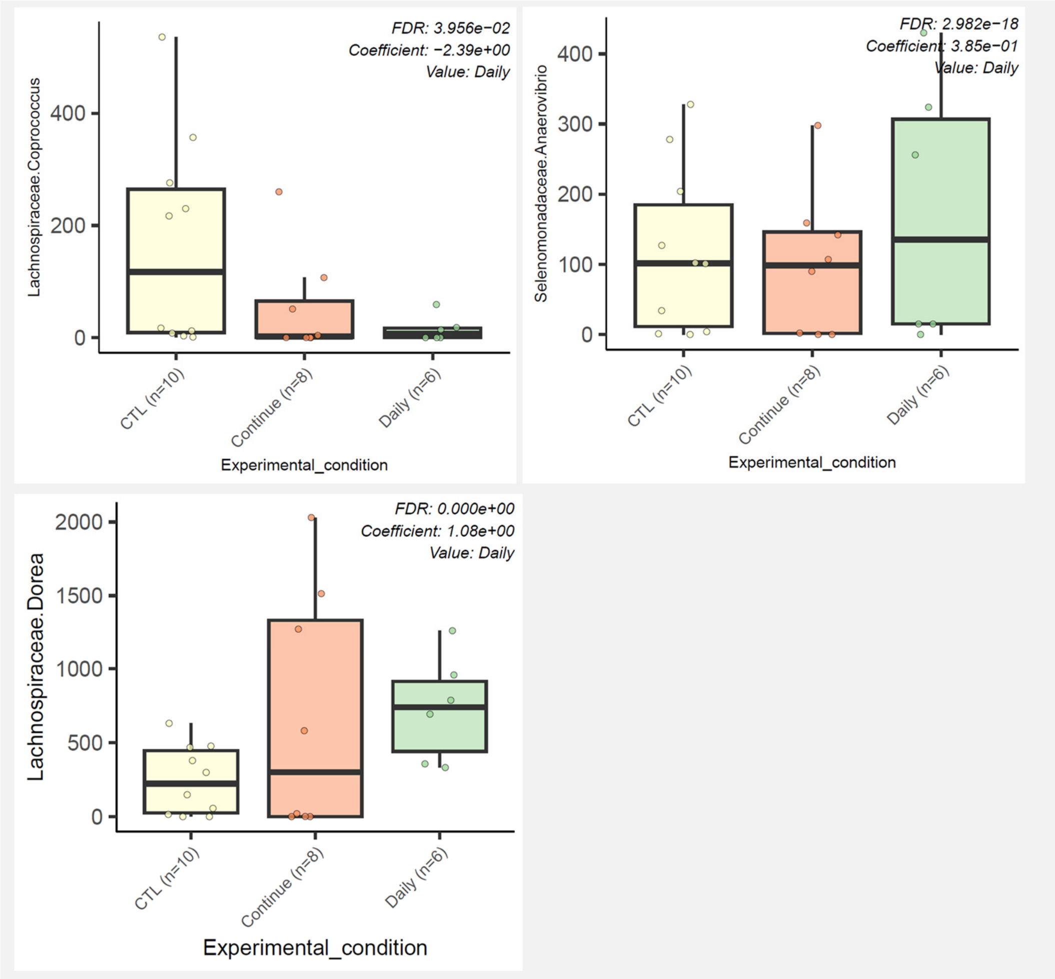

Supplementary Figure S8: Significant genera T120 for both treatment in comparison with the control analyzed by MaAslin2

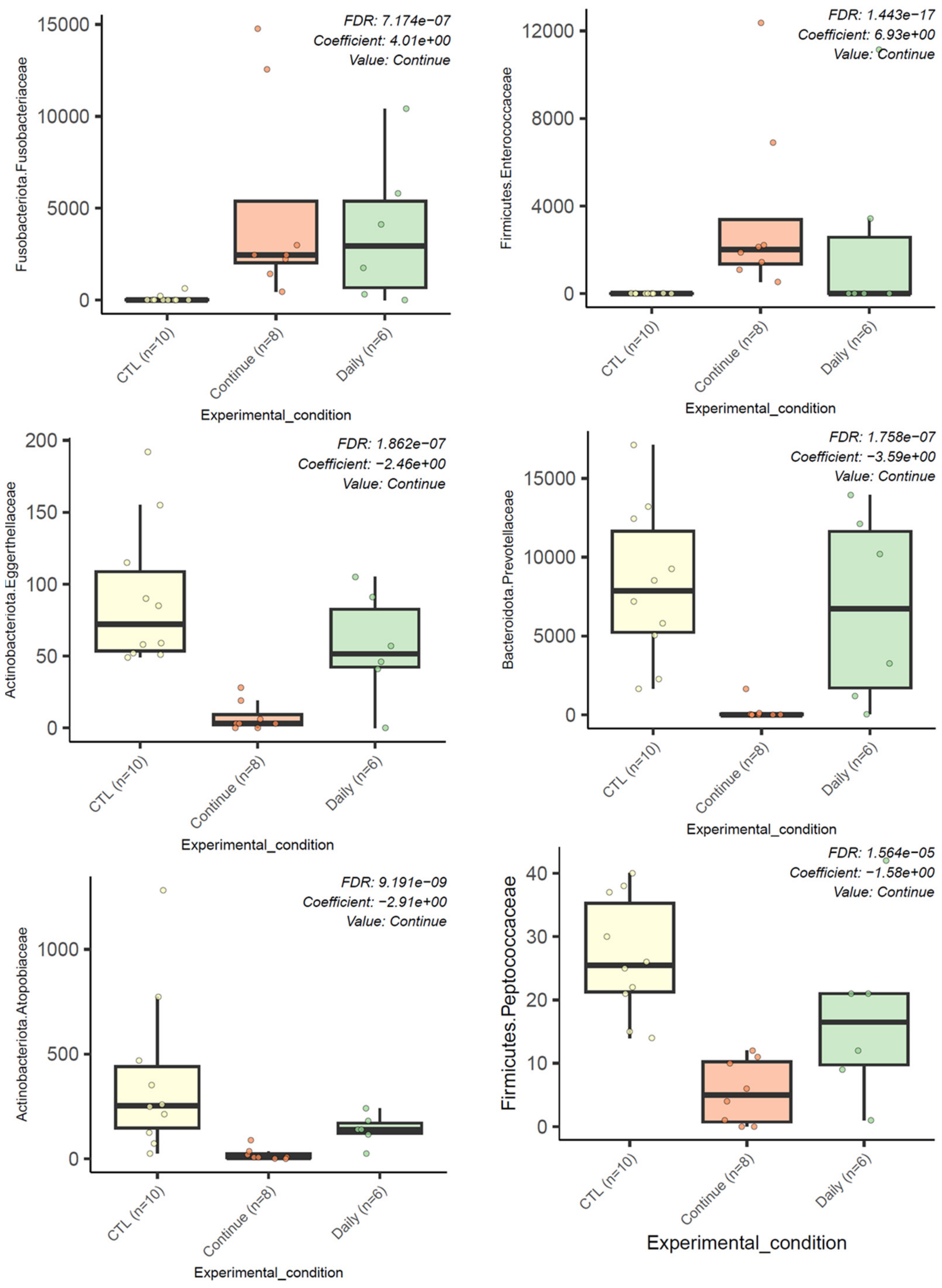
FDR: 7.174e−07
Coefficient: 4.01e+00
Value: Continue
Fusobacteriota.Fusobacteriaceae
15000
10000
5000
0
CTL (n=10)
Continue (n=8)
Daily (n=6)
Experimental_condition
FDR: 1.443e−17
Coefficient: 6.93e+00
Value: Continue
Firmicutes.Enterococcaceae
12000
8000
4000
0
CTL (n=10)
Continue (n=8)
Daily (n=6)
Experimental_condition
FDR: 1.862e−07
Coefficient: −2.46e+00
Value: Continue
Actinobacteriota.Eggerthellaceae
200
150
100
50
0
CTL (n=10)
Continue (n=8)
Daily (n=6)
Experimental_condition
FDR: 1.758e−07
Coefficient: −3.59e+00
Value: Continue
Bacteroidota.Prevotellaceae
15000
10000
5000
0
CTL (n=10)
Continue (n=8)
Daily (n=6)
Experimental_condition
FDR: 9.191e−09
Coefficient: −2.91e+00
Value: Continue
Actinobacteriota.Atopobiaceae
1000
500
0
CTL (n=10)
Continue (n=8)
Daily (n=6)
Experimental_condition
FDR: 1.564e−05
Coefficient: −1.58e+00
Value: Continue
Firmicutes.Peptococcaceae
40
30
20
10
0
CTL (n=10)
Continue (n=8)
Daily (n=6)
Experimental_condition

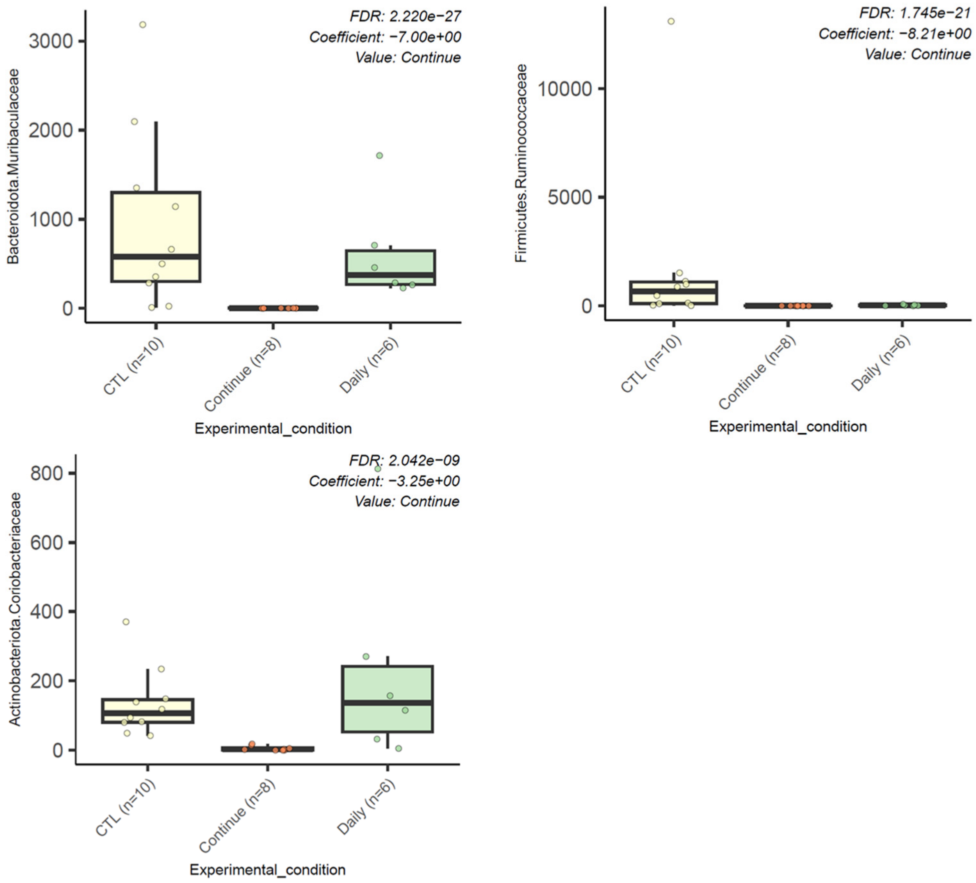

Supplementary Figure S9: Significant family T120 for both treatment in comparison with the control analyzed by MaAslin2

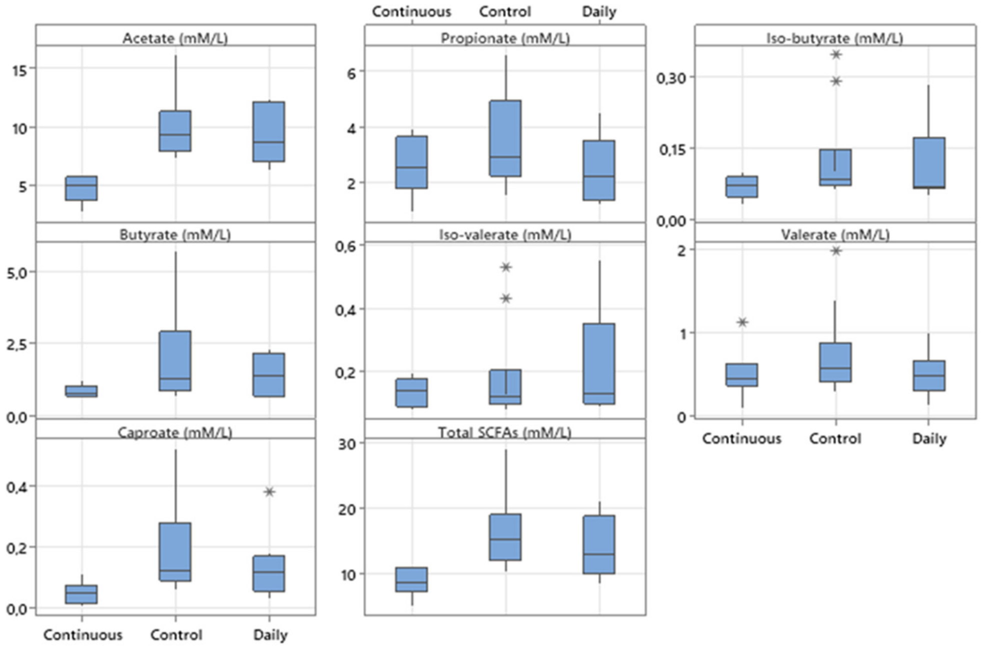

Supplementary Figure S10: SCFA analyzes in mM/mL

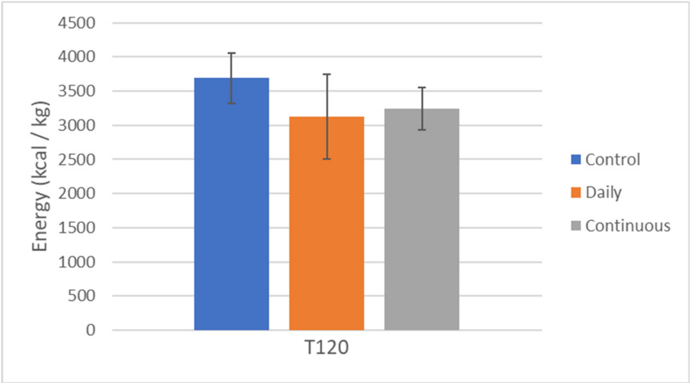

Supplementary Figure S11: Energy concentration in the sample at T120 in kcal/kg of bioreactor dry matter. The ANOVA test showed no significant result. (p = 0.14)

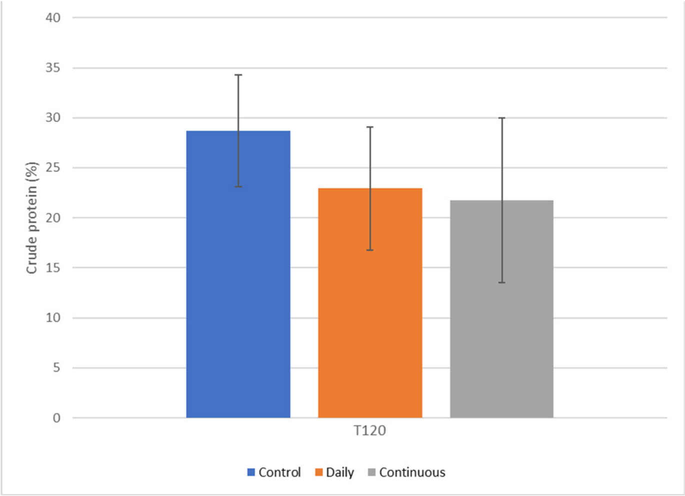

Supplementary Figure S12: Percentage of crude protein in the sample at T120. The ANOVA test showed no significant result. (p = 0.10)

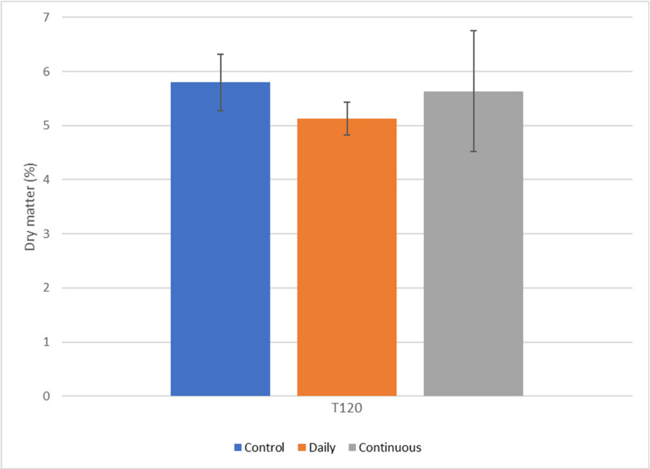

Supplementary Figure S13: Percentage of dry matter in the sample at T120. The ANOVA test showed no significant result. (p = 0.15)

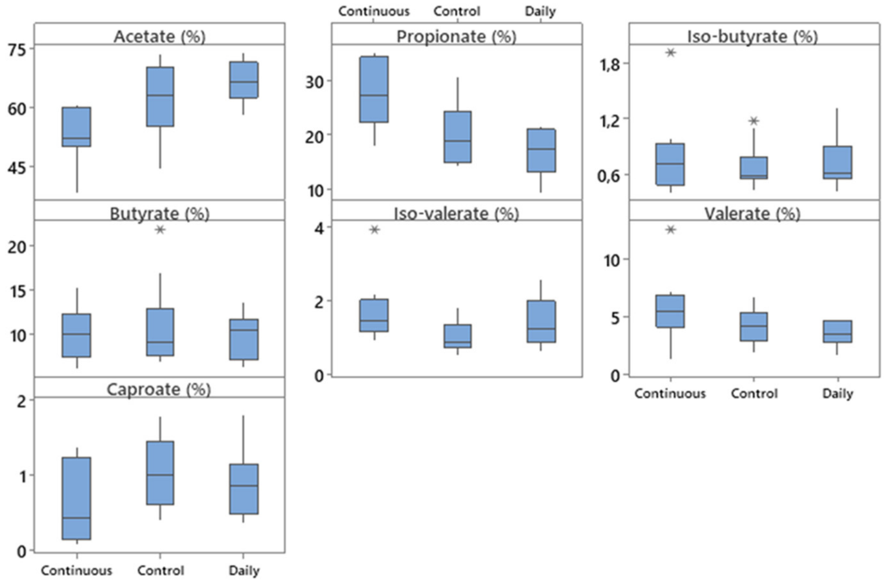

Supplementary Figure S14: SCFA analyzes in %

Supplementary Table S3: PCR reagent from Invitrogen SuperFi DNA polymerase kit

**Réactifs**	**Concentration finale**
SuperFi Buffer [5x]	1x
Enhancer [5x]	1x
dNTPs [10 mM]	0.2 mM
Forward primer [20 µM]	0.6 µM
Reverse primer [20 µM]	0.6 µM
BSA [20 mg/mL]	0.4 mg/mL
SuperFi TAQ polymerase [2 U/µL]	0.02 U/µL
H_2O PCR	N/A

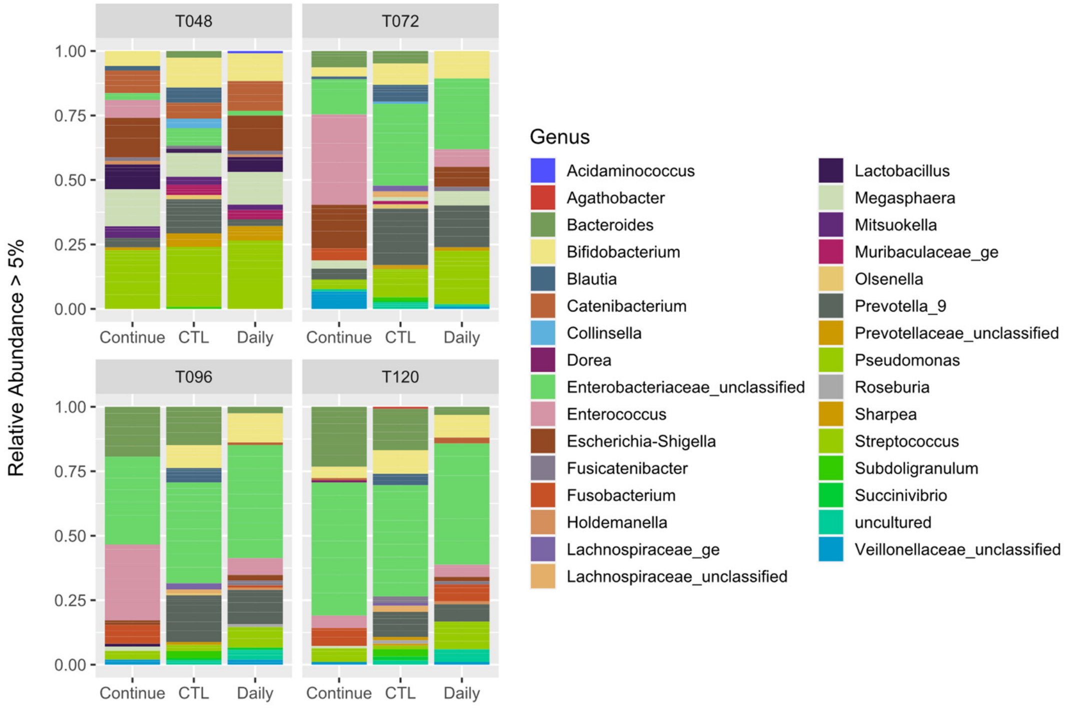

Supplementary Figure S15. Microbiota composition according to the time and probiotic use
